# The role of the *MfSWN1* in secondary wall growth and development

**DOI:** 10.3389/fpls.2026.1800907

**Published:** 2026-05-08

**Authors:** Lijia Xu, Yancen He, Ting Wan, Shenglong Hu, Sheng Wang, Meng Li, Zhiyong Chen

**Affiliations:** 1 College of Bioscience and Biotechnology, Hunan Agricultural University, Changsha, Hunan, China; 2 Hunan Engineering Laboratory of Miscanthus Ecological Application Technology, Hunan Agricultural University, Changsha, Hunan, China; 3 National Energy R&D Center for Non-food Biomass, Hunan Agricultural University, Changsha, Hunan, China; 4 Yuelushan Laboratory, Changsha, Hunan, China

**Keywords:** cellulose crystallinity index, cellulose degree of polymerization, lignocellulose, *MfSWN1*, secondary cell wall formation

## Abstract

**Background:**

The formation of secondary cell walls (SCWs) determines lignocellulosic composition and cell wall architecture. NAC transcription factors (*NAM*/*ATAF1*/*CUC2*) act as master regulators of the SCW transcriptional network. This study characterized *MfSWN1*, a *Miscanthus floridulus* NAC transcription factor homologous to NAC regulators of SCW synthesis, using *Arabidopsis thaliana* as a heterologous system.

**Methods:**

The wild-type (Col-0), the SCW-deficient double mutant *nst1–1 nst3-1*, *MfSWN1* complementation line (*MfSWN1*-OE/*nst1–1 nst3-1*), and *MfSWN1* overexpression line (*MfSWN1*-OX) were compared to profile the function of *MfSWN1*. Subsequent investigations focused on assessing developmental phenotypes, the expression of SCW marker genes, stem histology, cell wall composition, and cellulose structural properties [cellulose crystallinity index (CrI) and cellulose degree of polymerization (DP)] in stem tissues.

**Results:**

*MfSWN1* complementation partially rescued stem lodging and indehiscent silique phenotypes of *nst1–1 nst3-1*. Constitutive *MfSWN1* overexpression resulted in enlarged and wrinkled rosette leaves, delayed bolting, curved siliques, and enhanced lignification in stem tissues. *MfSWN1*-OE/*nst1–1 nst3–1* plants showed increased transcript levels of SCW-related genes (*MYB46*, *MYB83*, *CesA4*, *IRX7*, *IRX8*, *CCoAOMT1*, and *LAC4*) compared with the *nst1–1 nst3–1* mutant. In contrast, *MfSWN1*-OX plants showed increased expression of most of these genes relative to wild-type plants. Besides, *MfSWN1*-OX plants accumulated higher levels of cellulose, hemicellulose, and lignin, as well as increased CrI and DP than other genotypes.

**Conclusion:**

*MfSWN1* functions upstream in the NAC-MYB regulatory hierarchy, which can hasten coordinated SCW biosynthesis and modify cell wall structural organization in *Arabidopsis*. This study provides an evidence-based foundation for investigating *MfSWN1* and related NAC regulators in *Miscanthu*s.

## 1 Introduction

*Miscanthus floridulus* (*M. floridulus*) is a representative species of the genus *Miscanthus*, featured by exceptionally high biomass productivity and favorable cell wall properties. Unlike other *Miscanthus* species, *M. floridulus* exhibits high cellulose content, cellulose crystallinity index (CrI), and cellulose degree of polymerization (DP). Building upon these, *M. floridulus* is suitable for investigating lignocellulose formation and secondary cell wall (SCW) architecture. Furthermore, lignocellulose is the major structural component of plant cell walls, representing the most abundant renewable organic resource on Earth (Himmel et al., 2007). The composition and structural organization of lignocellulose are subjected to dynamic regulation of specific genes that control the biosynthesis and assembly of cell walls (Zhong and Ye, 2011). Changes in lignocellulosic composition and structural organization may produce significant impact on cell wall composition and architecture (Eom et al., 2015). Plant lignocellulose is mainly deposited in SCWs of fiber cells and vascular tissues. Among these, SCWs, consisting of cellulose, hemicellulose, and lignin primarily, can provide mechanical strength and structural support to plants (Zhu and Li, 2024). Given the complicated and recalcitrant structure of lignocellulose, understanding the genetic regulation of SCW biosynthesis is pivotal for clarifying the mechanism underlying cell wall composition and architecture (Gao et al., 2021). During SCW formation, NAC transcription factors (*NAM*/*ATAF1*/*CUC2*) generally act as master regulators to modulate the transcriptional network involved in lignocellulose biosynthesis (Xiao et al., 2018). *ND1*, *VND6*, *VND7*, *NST1*, and *NST2* are common members of the SCW NAC (SWN) family, usually functioning as upstream regulators of SCW formation. These transcription factors bind to a conserved cis-element known as the SWN binding element, an imperfect 19-bp palindromic sequence in the promoters of their target genes (Zhong et al., 2010b). Notably, the activation of NAC transcription factors may initiate a hierarchical regulatory cascade involving MYB transcription factors, and downstream structural genes responsible for the biosynthesis of cellulose, hemicellulose, and lignin.

According to functional studies in several plant species, SWN genes exert roles in SCW regulation. For example, the rice gene *OsSWN1*, a homolog of *Arabidopsis thaliana NST3*/*SND1*, serves as a key upstream regulator of SCW biosynthesis (Yoshida et al., 2013; Zhong et al., 2011). The *OsSWN1* promoter exhibits strong activity in tissues such as bundle sheath fibers and vascular tissues (culms, leaves, and mature anthers) undergoing SCW thickening. Constitutive expression of *OsSWN1* under the cauliflower mosaic virus 35S promoter in *Arabidopsis* triggers ectopic deposition of lignified SCWs in multiple tissues, including epidermal and cortical cells of inflorescence stems (Zhong and Ye, 2011). Meanwhile, under the control of the *Arabidopsis NST3*/*SND1* promoter, heterologous expression of *OsSWN1* can efficiently restore the formation of lignified SCWs in inflorescence fibers, and rescue the pendent stem phenotype in the *nst1 nst3* (*snd1*) mutant (Chai et al., 2015; Sakamoto et al., 2016). In addition, the *NST3*/*SND1* promoter-driven *OsSWN1* can promote lignified cell wall formation in the secondary xylem of transgenic *Arabidopsis* and poplar, without affecting plant growth (Sakamoto et al., 2016; Nayeri et al., 2022). Similarly, SWN homologs cloned from bamboo may function as strong transcriptional activators of SCW biosynthesis via their C-terminal activation domains, underlining their potential for engineering cell wall development (Sakamoto et al., 2022). Given the central regulatory role of NAC transcription factors in SCW formation, investigating SWN homologs from *Miscanthus* may provide valuable insights into the genetic regulation of lignocellulose biosynthesis. *Arabidopsis thaliana* has been extensively used as a model plant for studying the synthesis, assembly, transport, and modification of plant cell wall polymers, contributing to identifying numerous genes involved in SCW formation (Nakano et al., 2015; Taylor-Teeples et al., 2015). Functional characterization of these genes in model plants may provide critical data for understanding their roles in other plant species.

Accordingly, the present study was conducted to investigate the function of *MfSWN1*, a homolog of the rice gene *OsSWN1* cloned from *Miscanthus floridulus*, through heterologous expression in *Arabidopsis thaliana*. This study comparatively analyzed *MfSWN1* overexpression lines, mutant complementation lines, wild-type, and the SCW-deficient double mutant *nst1–1 nst3–1* to unveil effects of *MfSWN1* on plant morphology, SCW development, lignocellulosic composition, and cell wall structural properties. This work is anticipated to yield fresh insights into the interpretation of the regulatory role of *MfSWN1* in SCW biosynthesis, which may facilitate better understanding of SCW formation and cell wall structural organization in *Miscanthus* species.

## 2 Materials and methods

### 2.1 Plant materials

The *Arabidopsis thaliana* SCW-deficient double mutant *nst1–1 nst3-1* (T-DNA insertion line) and wild-type Columbia ecotype (Col-0) were obtained from the Arabidopsis Biological Resource Center. For the construction of complementation lines (*MfSWN1*-OE/*nst1–1 nst3-1*) and overexpression lines (*MfSWN1*-OX), the *MfSWN1* coding sequence was cloned into the pEZR(K)-LK binary vector under the control of the 35S promoter, generating the pEZR(K)-LK-*Pro.35S::MfSWN1* construct. The recombinant plasmid was introduced into *Agrobacterium tumefaciens* EHA105, which was then used for plant transformation using the floral dip method. In terms of complementation, the construct was transformed into the *nst1–1 nst3–1* mutant background to construct *MfSWN1*-OE/*nst1–1 nst3–1* plants. For overexpression lines, the same construct was transformed into Col-0 plants to obtain *MfSWN1*-OX plants. Transgenic plants were screened and propagated to the T3 generation, with the stable lines preserved within our laboratory. *Arabidopsis* were grown in a controlled chamber at 22 °C under long-day conditions (16 h-8 h light/dark cycle).

### 2.2 Identification of *MfSWN1*-OE/*nst1–1 nst3–1* and *MfSWN1*-OX *Arabidopsis thaliana*

At approximately 4–5 weeks after germination, genomic DNA was extracted from the T3-generation plants of *MfSWN1*-OX and *MfSWN1*-OE/*nst1–1 nst3-1*. In order to verify transgene insertion, polymerase chain reaction (PCR) amplification was performed using *MfSWN1*-specific primers. The yielded products were analyzed by agarose gel electrophoresis.

### 2.3 Analysis of phenotypic differences

Throughout the growth period, *Arabidopsis* (Col-0, *MfSWN1*-OE/*nst1–1 nst3-1*, *MfSWN1*-OX, and *nst1–1 nst3-1*) were monitored to collect and record corresponding phenotypic characteristics. Morphological traits, including rosette leaf morphology, bolting time, stem growth, and silique development, were observed and documented using digital photography.

### 2.4 Gene expression analysis

Stem tissues were collected from 4-5-week-old *Arabidopsis* (Col-0, *MfSWN1*-OE/*nst1–1 nst3-1*, *MfSWN1*-OX, and *nst1–1 nst3-1*). Using the Plant RNA Kit R6827 (OMEGA) as instructed, total RNA was extracted by collecting about 100 mg of stem tissue from the basal region of each plant. With the verification of RNA quality and concentration, the first-strand cDNA was synthesized using total RNA as the template. Quantitative real-time PCR (qRT-PCR) was performed using All-Style Gold qPCR Mix on an Applied Biosystems 7300 Fast Real-Time PCR System. The PCR program was summarized as follows: denaturation at 94 °C for 30 s initially, followed by 45 cycles of 94 °C for 5 s and 60 °C for 31 s. The amplification specificity was validated by employing a melting curve analysis (95 °C for 15 s, 60 °C for 1 min, and 95 °C for 15 s). Relative expression levels of several genes involved in SCW biosynthesis were calculated using the 2^−ΔΔCt^ method. The specific genes included the MYB transcription factors *MYB46* and *MYB83*, the cellulose synthase gene *CesA4*, the xylan biosynthesis genes *IRX7* and *IRX8*, the lignin biosynthesis gene *CCoAOMT1*, and the lignin polymerization gene *LAC4*.

### 2.5 Cytologic observations

#### 2.5.1 Stem section preparation

The Col-0, *MfSWN1*-OE/*nst1–1 nst3-1*, *MfSWN1*-OX plants, and *nst1–1 nst3–1* mutants (4-5-week-old) were collected around 2–3 cm above the base of the stem. Stem segments were manually sectioned using a sharp razor blade, and the samples during sectioning were stabilized using fresh carrot tissue as a supporting matrix. After transfer into distilled water, these stem segments transverse sections were mounted on glass slides. Finally, all these stem segments transverse sections were covered with a coverslip for microscopic observation.

#### 2.5.2 Histological staining for lignin

The deposition of lignin was detected using the phloroglucinol-hydrochloric acid (HCl) staining. Briefly, about 50 μL of lignin acidifying solution was added to the sections approximately 2 min of incubation to allow reagent penetration. After that, the stained sections, following processing with 50 μL of phloroglucinol staining solution, were observed under a bright-field microscope.

#### 2.5.3 Fluorescent staining of cellulose

The Calcofluor White fluorescent staining was further employed to examine the cellulose distribution in stem segments transverse sections. A drop of Calcofluor White stain and a drop of 10% potassium hydroxide were applied to the sections for incubation to achieve uniform staining. The stained samples were covered with a coverslip for further observation under blue illumination using a fluorescence microscope.

### 2.6 Determination of lignocellulosic composition

#### 2.6.1 Preparation of alcohol-insoluble residue via Soxhlet extraction

The 6-week-old *Arabidopsis* (Col-0, *MfSWN1*-OE/*nst1–1 nst3-1*, *MfSWN1*-OX, and *nst1–1 nst3-1*) were harvested to collect stem tissues from the base of the rosette. Four different *Arabidopsis* samples were dried at 60 °C to constant weight, ground into powder, and passed through a standard test sieve with a mesh size of 0.425 mm. *Arabidopsis* powder sieved using standard test sieves ( c) was placed into filter paper thimbles for the removal of soluble compounds via Soxhlet extraction with 75% ethanol. The extracted samples were dried at 105 °C to constant weight ( d), and the residue was recorded for subsequent compositional analysis. In addition, the moisture content before ( a) and after ( b) extraction was determined using a moisture analyzer.

#### 2.6.2 Acid hydrolysis

Approximately 0.3 g of the Soxhlet extraction sample from 2.6.1 was added with 3 mL of 72% sulfuric acid (H_2_SO_4_) after transferring into the 100 mL pressure bottles. The samples were mixed thoroughly to ensure complete impregnation, followed by 1 h of incubation at 30 °C with shaking at 150 rpm. After the initial hydrolysis, the sulfuric acid concentration was diluted to 4% by supplementing 84 mL of distilled water. Finally, the samples were autoclaved at 121 °C for 30 min to complete hydrolysis.

#### 2.6.3 Filtration and hydrolysate preparation

The hydrolysis products obtained from *Section 2.6.2* were filtered through a glass fiber crucible using a vacuum filtration system to collect the filtrate for sugar analysis. Meanwhile, the residue in the crucible was washed with distilled water repeatedly until neutral pH was reached. Furthermore, to determine soluble components, a portion of the filtrate from crucible filtration was diluted and analyzed spectrophotometrically at 320 nm. Another aliquot was filtered from a 0.45-μm hydrophobic syringe filter and injected into a high-performance liquid chromatography system to quantify released saccharides. The residue and crucible were dried at 105 °C to constant weight and weighed ( $G_{1}$). Then, the crucible was transferred to a muffle furnace at 550 °C for 3 h for ashing to yield the final weight, which was recorded as $G_{2}$.

#### 2.6.4 Calculation of cell wall components

The extraction residue rate was calculated as:


$$X=\frac{(1-b)\times d}{(1-a)\times c}\times 100$$

where X is the extraction residue rate (%), a is the moisture content before extraction, b is the moisture content after extraction, c is the initial sample weight (g), and d is the sample weight after extraction (g).

##### 2.6.4.1 Lignin content


$$L_{1}=\frac{U\times V\times n\times X\times 100}{\iota \times \epsilon \times W\times (1-b)}$$

$$L_{2}=\frac{((G_{1}-G_{0})-(G_{2}-G_{0}))\times X\times 100}{W\times (1-b)}$$

where $L=L_{1}+L_{2}$, L is the lignin content (%), U is the absorbance at 320 nm, V is the hydrolysate volume (L), n is the dilution factor, ι is the optical path length (cm), ϵ is the absorption coefficient [L/(g·cm)], W is the sample weight (g), $G_{0}$ is the empty crucible weight (g), $G_{1}$ is the crucible plus residue weight (g), and $G_{2}$ is the crucible plus ash weight (g). The other variables are as defined above.

##### 2.6.4.2 Cellulose content


$$Y_{1}=\frac{(c_{1}+c_{2}\times 1.0526)\times 0.90\times V\times X\times 100}{W\times (1-b)\times R_{1}}$$

where $Y_{1}$ is the cellulose content (%), $c_{1}$ is the glucose concentration (mg/mL), $c_{2}$ is the cellobiose concentration (mg/mL), and $R_{1}$ is the glucose recovery coefficient. The other variables are as defined above.

##### 2.6.4.3 Hemicellulose content


$$Y_{4}=\frac{c_{3}\times 0.88\times V\times X\times 100}{W\times (1-b)\times R_{2}}$$

$$Y_{5}=\frac{c_{4}\times 0.88\times V\times X\times 100}{W\times (1-b)\times R_{3}}$$

where $Y_{2}=Y_{4}+Y_{5}$, $\;Y_{2}$ is the hemicellulose content (%), $c_{3}$ is the xylose concentration (mg/mL), $c_{4}$ is the arabinose concentration (mg/mL), and $R_{2}$ and $R_{3}$ are the recovery coefficients of xylose and arabinose, respectively. The other variables are as defined above.

##### 2.6.4.4 Pectin content


$$Y_{3}=\frac{c_{5}\times 0.92\times V\times X}{W\times (1-b)\times R_{4}}\times 100$$

where $Y_{3}$ is the pectin content (%), $c_{5}$ is the galacturonic acid concentration (mg/mL), $R_{4}$ is the recovery coefficients of galacturonic acid. The other variables are as defined above.

### 2.7 Determination of lignocellulose crystallinity

An appropriate amount of the Soxhlet extraction sample from 2.6.1 was evenly spread onto the sample holder and compacted prior to the analysis. Following the method proposed by Segal et al (Segal et al., 1959). Cellulose CrI was measured using an X-ray powder diffractometer (XRD-6000, Shimadzu, Japan). The measurement was conducted under conditions of Cu Kα radiation (λ = 0.15406 nm), tube voltage of 40 kV, tube current of 30 mA, scanning speed of 5° min^-^¹, step size of 0.02°, divergence slit of 1°, receiving slit of 0.3 mm, scattering slit of 1°, and scanning range 2^θ^ = 10–40°.


$$\text{CrI}(\%)=((I_{200}-I_{am})/I_{200})\times 100$$

where $I_{200}$, and $I_{am}$ represent the diffraction intensities of lignocellulose in the crystalline region and the amorphous region, respectively. $I_{200}$, and $I_{am}$ correspond to the maximum peak near 2^θ^ ≈ 22.5° and 2^θ^ ≈ 18°, respectively.

### 2.8 Determination of cellulose degree of polymerization

0.100 g of the Soxhlet extraction sample from 2.6.1 ( $W_{0}$) was weighed and transferred into a 50 mL centrifuge tube, with 20 mL of 0.5 mol/L COPPER-ETHYLENEDIAMINE COMPLEX solution added subsequently. Another 20 mL COPPER-ETHYLENEDIAMINE COMPLEX solution was used as the control. The samples were subjected to 2 h of incubation in a shaker at 25 °C and 150 rpm to dissolve the lignocellulose. After that, the obtained solutions were centrifuged for 5 min at 2,810 rpm to obtain the supernatant, which was transferred into another centrifuge tube. Both the sample solution and the control solvent were equilibrated in a thermostatic water bath at 25 °C for 30 min. The next step was the recording of the flow time of the solution between the upper and lower marks of an Ubbelohde Viscometer. Among them, the average flow time of the sample solution and the control solvent was recorded as T and $T_{0}$, respectively. Each measurement was repeated twice to take the mean values for further calculations. The precipitate in the centrifuge tube was washed repeatedly with distilled water by centrifugation at 2,810 rpm until the solution became colorless. Eventually, the precipitate after rinsing with distilled water was dried at 105 °C to constant weight to weigh and record the final sample weight ( W).

### 2.9 Polymerization calculations

#### 2.9.1 Relative viscosity of the sample


$${\text{η}}_{\text{rel}}=\frac{T}{T_{0}}$$

where ${\text{η}}_{\text{rel}}$ is the relative viscosity, T is the average flow time of the sample solution (s), $T_{0}$ is the average flow time of the control solvent (s).

#### 2.9.2 Sample solution concentration


$$\text{C}=\frac{W_{0}-W}{V}$$

where C is the concentration of the sample solution (g/mL), $W_{0}$ is the mass of the sample before dissolution (g), W is the mass of the undissolved residue (g), and V is the volume of CuEN solvent (mL; 20 mL in this study).

#### 2.9.3 Intrinsic viscosity of the sample solution

According to the relative viscosity ( ${\text{η}}_{\text{rel}}$) of the sample solution and the solvent, the corresponding **[** η**]**·C value was obtained from Appendix Table. Combined with the sample concentration (C), the intrinsic viscosity **[** η**]** of the sample solution was determined.

#### 2.9.4 Degree of polymerization


$$DP^{0.905}=0.75\cdot [\text{η}]$$

where DP is the degree of polymerization of cellulose and **[** η**]** is the intrinsic viscosity of the sample solution.

## 3 Results

### 3.1 Identification of *MfSWN1*-OE/*nst1–1 nst3–1* and *MfSWN1*-OX lines

**Figure 1:**
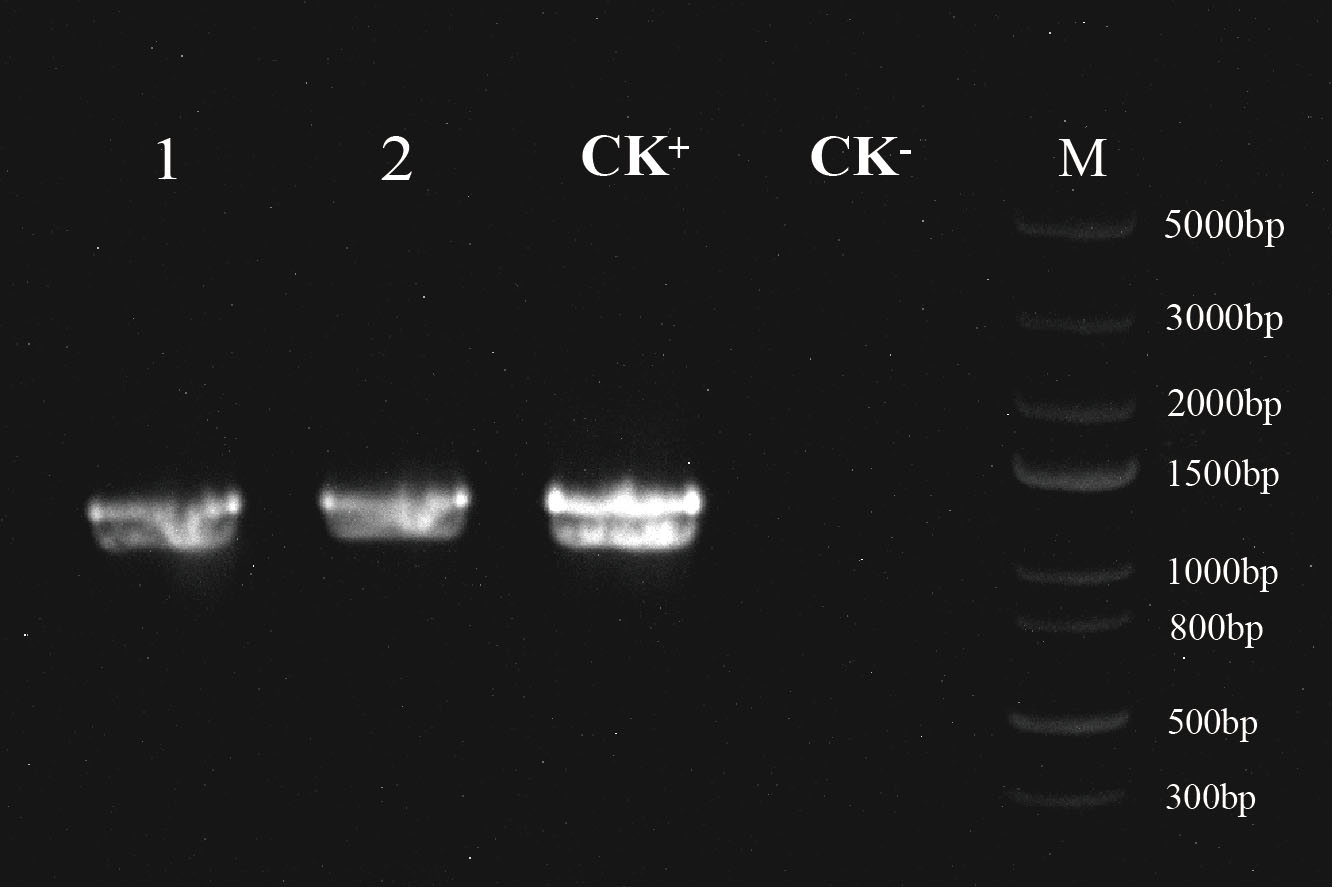
Identification of transgenic *Arabidopsis thaliana* plants by PCR. M, DL5000 DNA marker; lane 1, *MfSWN1*-OX plants; lane 2, *MfSWN1*-OE/*nst1–1 nst3–1* plants; CK+, positive control; CK−, negative control. The expected size of the PCR fragment is 1,236 bp.

Based on PCR analysis using *MfSWN1*-specific primers, *MfSWN1* transgene was presented in both *MfSWN1*-OE/*nst1–1 nst3–1* and *MfSWN1*-OX plants. The expected PCR fragment (1,236 bp), absent in the negative control, was detected in the DNA from transgenic lines, indicating that the *MfSWN1* gene was successfully introduced into these lines (**Figure 1**).

### 3.2 Phenotypic characterization of Col-0, *MfSWN1*-OE/*nst1–1 nst3-1*, *MfSWN1*-OX and *nst1–1 nst3–1* lines

**Figure 2:**
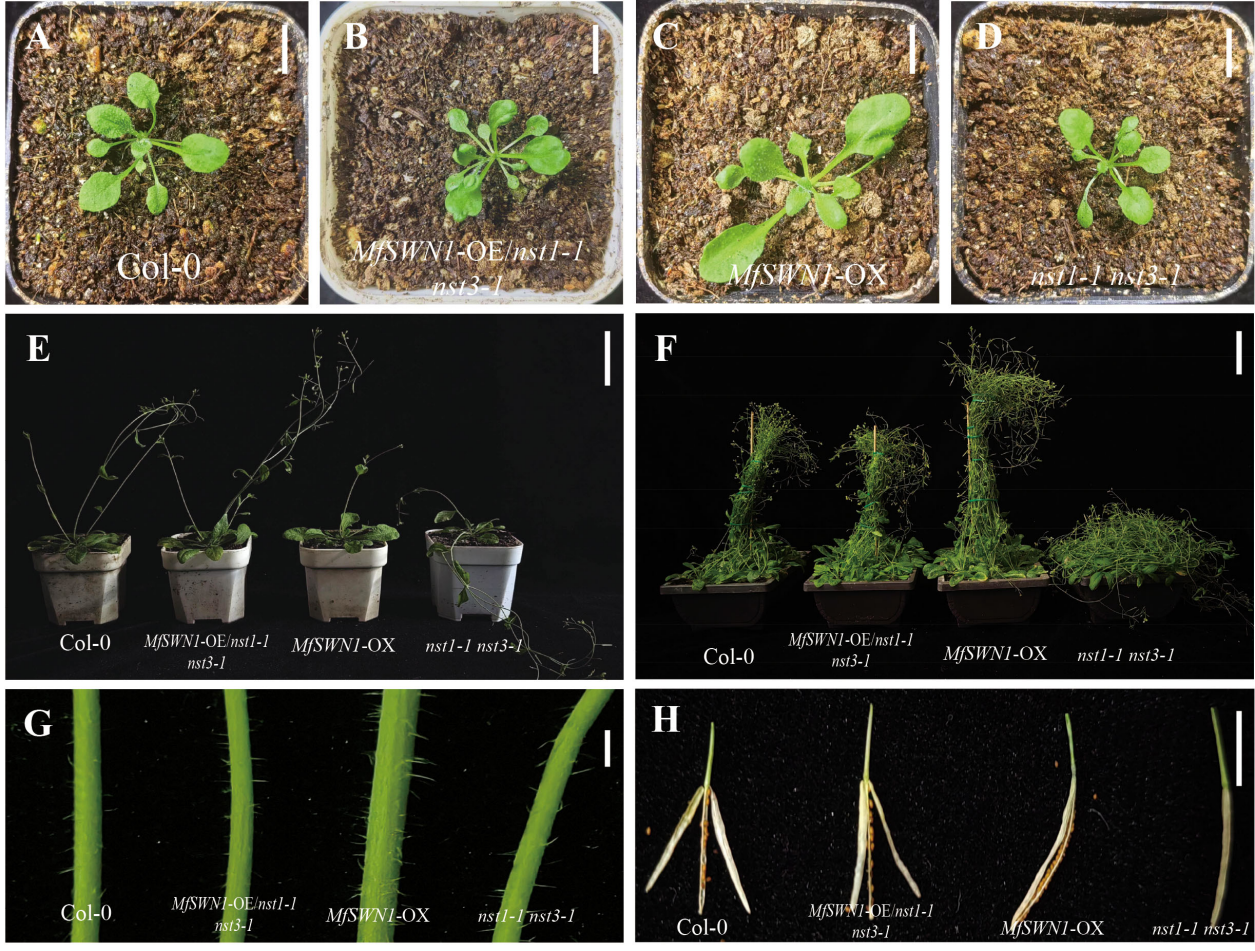
Phenotypic comparison of *Arabidopsis thaliana*. Col-0 **(A)**, *MfSWN1*-OE/*nst1–1 nst3-1* **(B)**, *MfSWN1*-OX **(C)**, and *nst1–1 nst3-1* **(D)**. **(A–D)** Plant morphology at 3 weeks after germination. Scale bar = 3 cm. **(E)** Plant morphology at 6 weeks. Scale bar = 5 cm. **(F)** Plant morphology at 7 weeks. Scale bar = 5 cm. **(G)** Main stem morphology at 7 weeks. Scale bar = 2 mm. **(H)** Mature silique morphology. Scale bar = 1 cm.

The phenotypic differences among the four *Arabidopsis* plants constructed were examined during plant development. At the third week, *MfSWN1*-OX plants exhibited markedly enlarged and wrinkled rosette leaves compared with Col-0 plants. And *MfSWN1*-OE/*nst1–1 nst3–1* plants showed a morphology more similar to that of Col-0 plants than to that of the *nst1–1 nst3–1* mutant (**Figures 2A–D**). Furthermore, bolting was initiated in Col-0 plants, *MfSWN1*-OE/*nst1–1 nst3-1*, and *nst1–1 nst3–1* mutants at around the fourth week, whereas delayed bolting was observed in *MfSWN1*-OX plants until approximately the fifth week (**Figure 2E**). Moreover, *MfSWN1*-OX plants grew rapidly after bolting. By the seventh week, beyond higher plant height than the other genotypes, *MfSWN1*-OX plants also exhibited increased main stem diameter (**Figures 2F, G**). In addition, in the mature siliques, *MfSWN1*-OX plants developed strongly curved siliques, whereas *MfSWN1*-OE/*nst1–1 nst3–1* plants showed partial restoration of the indehiscent silique phenotype of the *nst1–1 nst3–1* mutants(**Figure 2H**).

### 3.3 Transcriptional profiling of secondary wall biosynthetic genes

**Figure 3:**
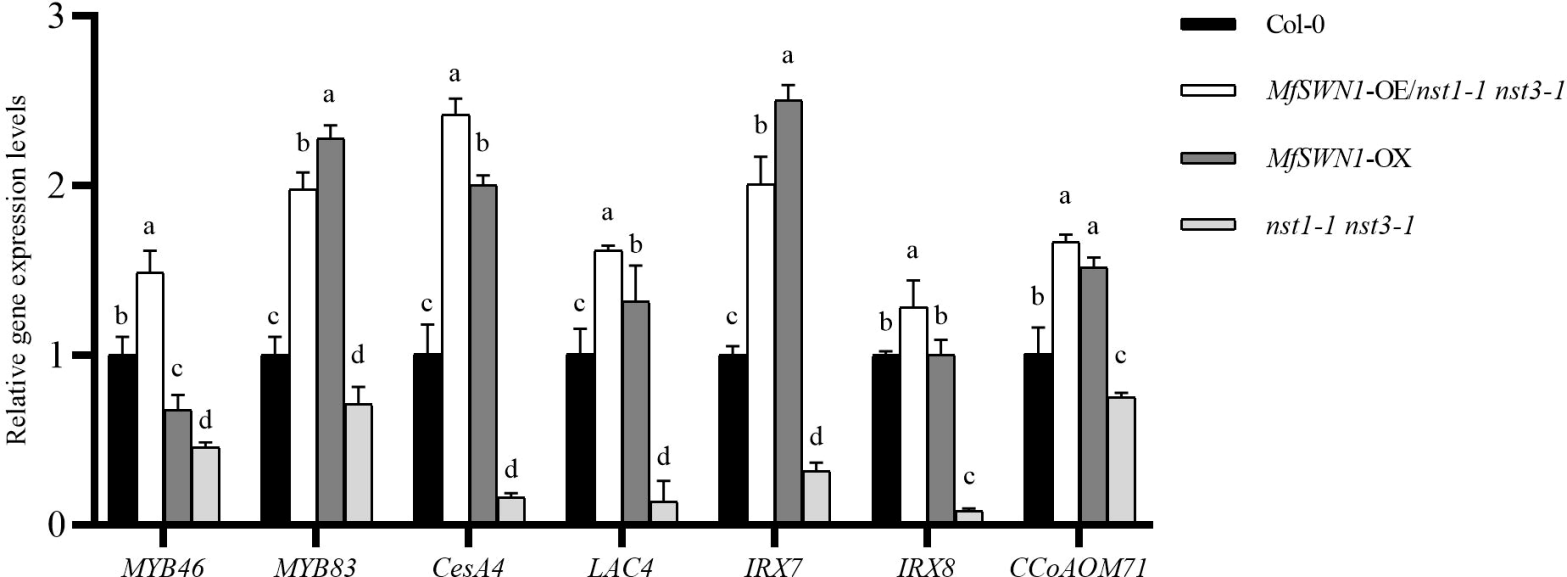
Expression levels of SCW formation-associated genes (*MYB46*, *MYB83*, *CesA4*, *LAC4*, *IRX7*, *IRX8*, and *CCoAOMT1*) in the four *Arabidopsis thaliana* plants constructed in this study (Col-0, *MfSWN1*-OE/*nst1–1 nst3-1*, *MfSWN1*-OX, and *nst1–1 nst3-1*). Different lowercase letters indicate significant differences among genotypes (*P* < 0.05).

The transcript abundance of key genes involved in SCW biosynthesis was quantified in the four *Arabidopsis* plants constructed. As expected, compared with the Col-0 plants, the *nst1–1 nst3–1* mutant was detected with significantly depleted transcripts of *MYB46*, *MYB83*, *CesA4*, *LAC4*, *IRX7*, *IRX8*, and *CCoAOMT1* (**Figure 3**). However, the introduction of *MfSWN1* (*MfSWN1*-OE/*nst1–1 nst3-1*) robustly restored the expression of these downstream targets to levels comparable to, or exceeding, those in the Col-0 plants. Concurrently, relative to Col-0 plants, the *MfSWN1*-OX plants showed a general, albeit gene-dependent, upregulation of SCW-related transcripts. Thus, the *MfSWN1*-OE/*nst1–1 nst3–1* plants could functionally complement the transcriptional deficiency of the mutants, while the *MfSWN1*-OX plants demonstrated an inherent capacity of the gene to drive transcriptional activation in the wild-type context. Collectively, these data provided compelling evidence that *MfSWN1* successfully activated the magnitude of induction in a gene-dependent manner. These findings supported the presence of a downstream SCW biosynthetic cascade.

### 3.4 Cytological characterization of stem tissues

**Figure 4:**
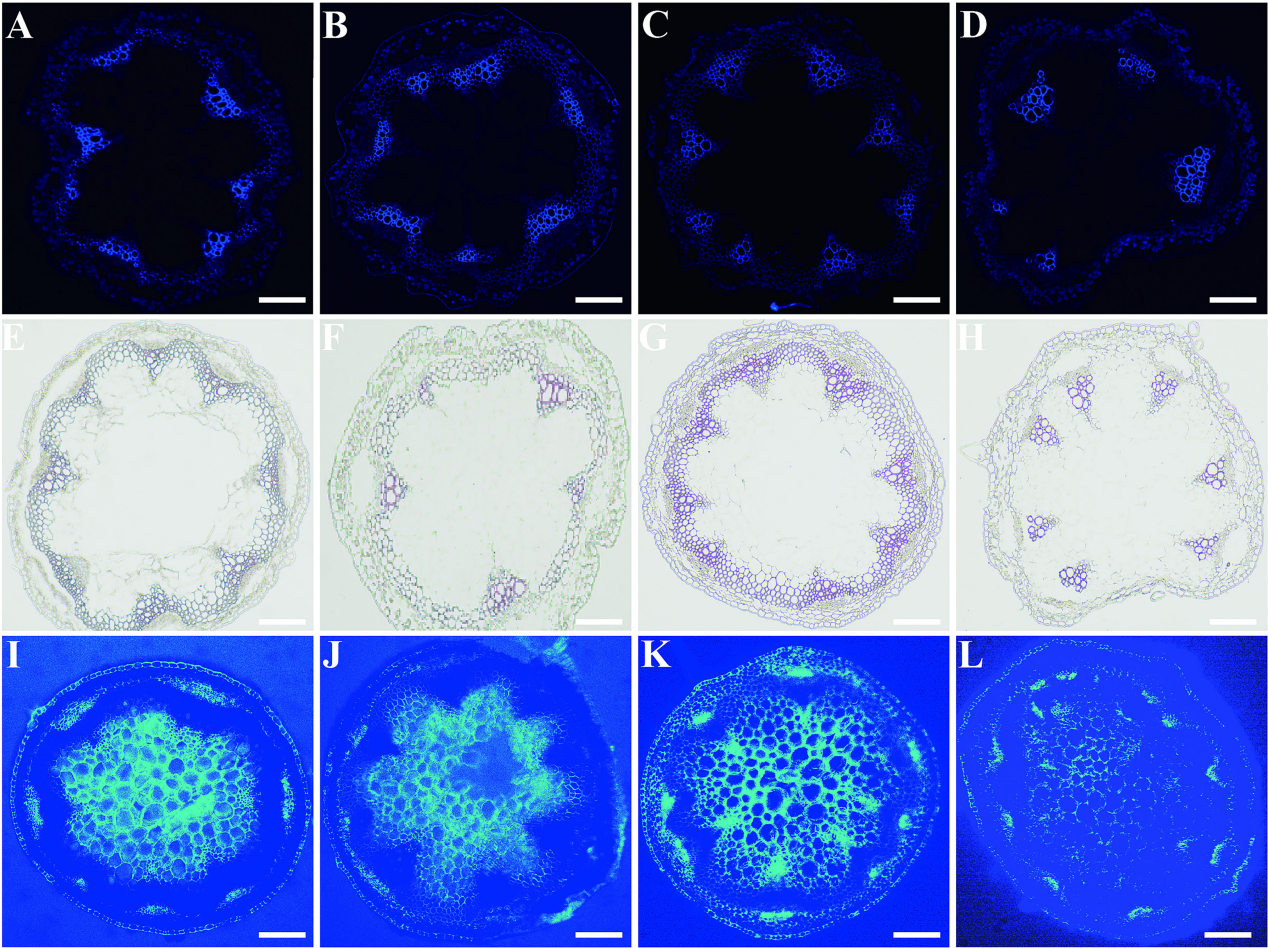
The deposition of lignin and cellulose in stem cross-sections of *Arabidopsis thaliana*. Col-0 **(A, E, I)**, *MfSWN1*-OE/*nst1–1 nst3-1* **(B, F, J)**, *MfSWN1*-OX **(C, G, K)**, and *nst1–1 nst3-1* **(D, H, L)**. Panels **(A–D)** show cell wall autofluorescence, **(E–H)** display lignin staining, and **(I–L)** represent cellulose staining. Scale bars = 500 μm.

Stem sections from 4-5-week-old Col-0, *MfSWN1*-OE/*nst1–1 nst3-1*, *MfSWN1*-OX plants, and *nst1–1 nst3–1* mutants were analyzed via fluorescence microscopy. Under blue-light excitation, the *nst1–1 nst3–1* mutants showed an absence of lignified SCW deposition in the interfascicular fiber region. In contrast, Col-0, *MfSWN1*-OE/*nst1–1 nst3-1*, and *MfSWN1*-OX plants displayed distinct lignin autofluorescence signals (**Figures 4A–D**). This proved that introducing *MfSWN1* into the mutant background successfully restored lignified SCW formation. Lignin deposition was further verified via phloroglucinol-HCl staining. Notably, *MfSWN1*-OX plants developed thicker SCW compared with the Col-0 and *MfSWN1*-OE/*nst1–1 nst3–1* plants(**Figures 4E–H**). Additionally, cellulose deposition was visualized using Calcofluor White staining. Under identical exposure conditions, the *nst1–1 nst3–1* mutants exhibited the weakest fluorescence, whereas the Col-0 and *MfSWN1*-OE/*nst1–1 nst3–1* plants displayed comparable signal intensities. Notably, the *MfSWN1*-OX plants showed the strongest fluorescence, indicative of enhanced cellulose deposition in the SCW(**Figures 4I–L**). Collectively, these findings demonstrate that *MfSWN1* promotes the accumulation of both lignin and cellulose in SCW.

### 3.5 Lignocellulosic composition analysis

**Figure 5:**
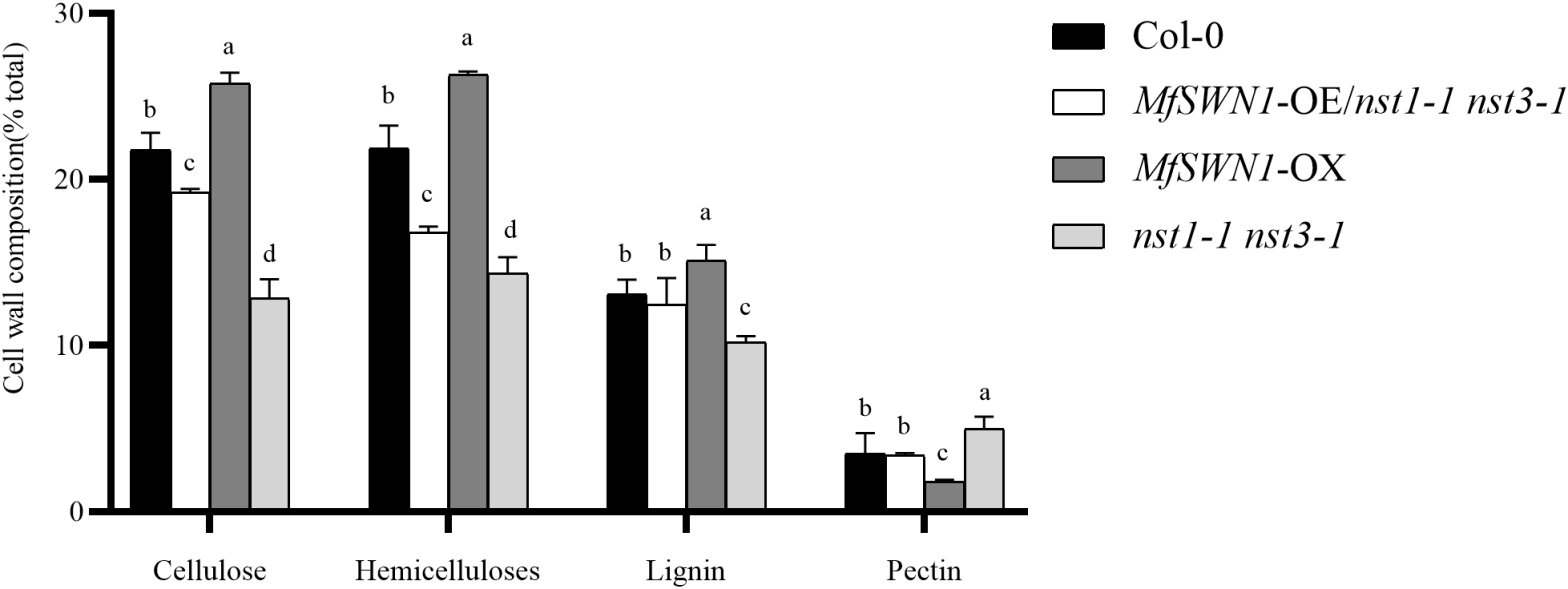
Determination of major cell wall components (cellulose, hemicellulose, lignin, and pectin) in the four *Arabidopsis thaliana* plants constructed in this study (Col-0, *MfSWN1*-OE/*nst1–1 nst3-1*, *MfSWN1*-OX, and *nst1–1 nst3-1*). Different letters indicate significant differences among genotypes (*P* < 0.05).

To evaluate lignocellulosic composition, the contents of cellulose, hemicellulose, and lignin were quantified in Col-0, *MfSWN1*-OE/*nst1–1 nst3-1*, *MfSWN1*-OX plants, and *nst1–1 nst3–1* mutants. The analysis revealed that *MfSWN1*-OX plants accumulated significantly higher levels of these three structural components compared to Col-0 and *MfSWN1*-OE/*nst1–1 nst3–1* plants. Furthermore, both the Col-0 and *MfSWN1*-OE/*nst1–1 nst3–1* plants exhibited significantly greater lignocellulosic contents than *nst1–1 nst3–1* mutants (**Figure 5**).

These findings indicate that *MfSWN1* positively regulates the accumulation of major secondary wall components. Conversely, pectin content displayed an inverse pattern, with levels increasing in the order of *MfSWN1*-OX < *MfSWN1*-OE/*nst1–1 nst3-1* < Col-0 < *nst1–1 nst3-1*.

### 3.6 Lignocellulosic structural properties of cell walls

**Figure 6:**
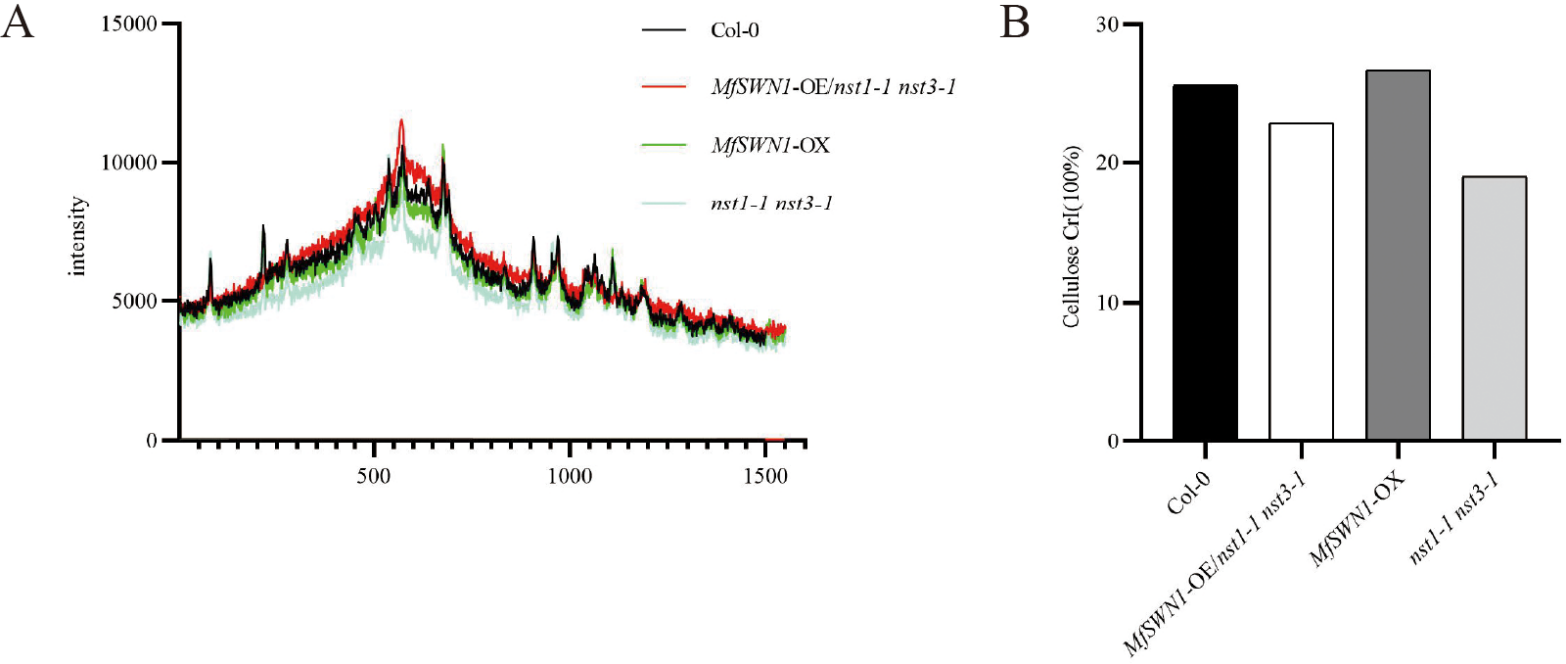
Determination of the CrI value of crude cell walls from the stems of 6-week-old *Arabidopsis* plants. **(A)** X-ray diffraction patterns of crude cell walls isolated from Col-0, *MfSWN1*-OX, *MfSWN1*-OE/*nst1–1 nst3-1*, and *nst1–1 nst3–1* plants. **(B)** CrI values calculated from the X-ray diffraction patterns.

To investigate the structural properties of lignocellulose, Col-0, *MfSWN1*-OE/*nst1–1 nst3-1*, *MfSWN1*-OX plants, and *nst1–1 nst3–1* mutants were analyzed via X-ray diffraction (XRD) and viscosity-based polymerization measurements. While the XRD patterns indicated a similar overall cellulose crystal structure across the four genotypes (**Figure 6A**), the cellulose CrI varied significantly. Specifically, the CrI values decreased in the following order: *MfSWN1*-OX > Col-0 > *MfSWN1*-OE/*nst1–1 nst3-1* > *nst1–1 nst3-1* (**Figure 6B**). These findings demonstrate that *MfSWN1* expression is positively correlated with enhanced cellulose crystallinity.

**Figure 7:**
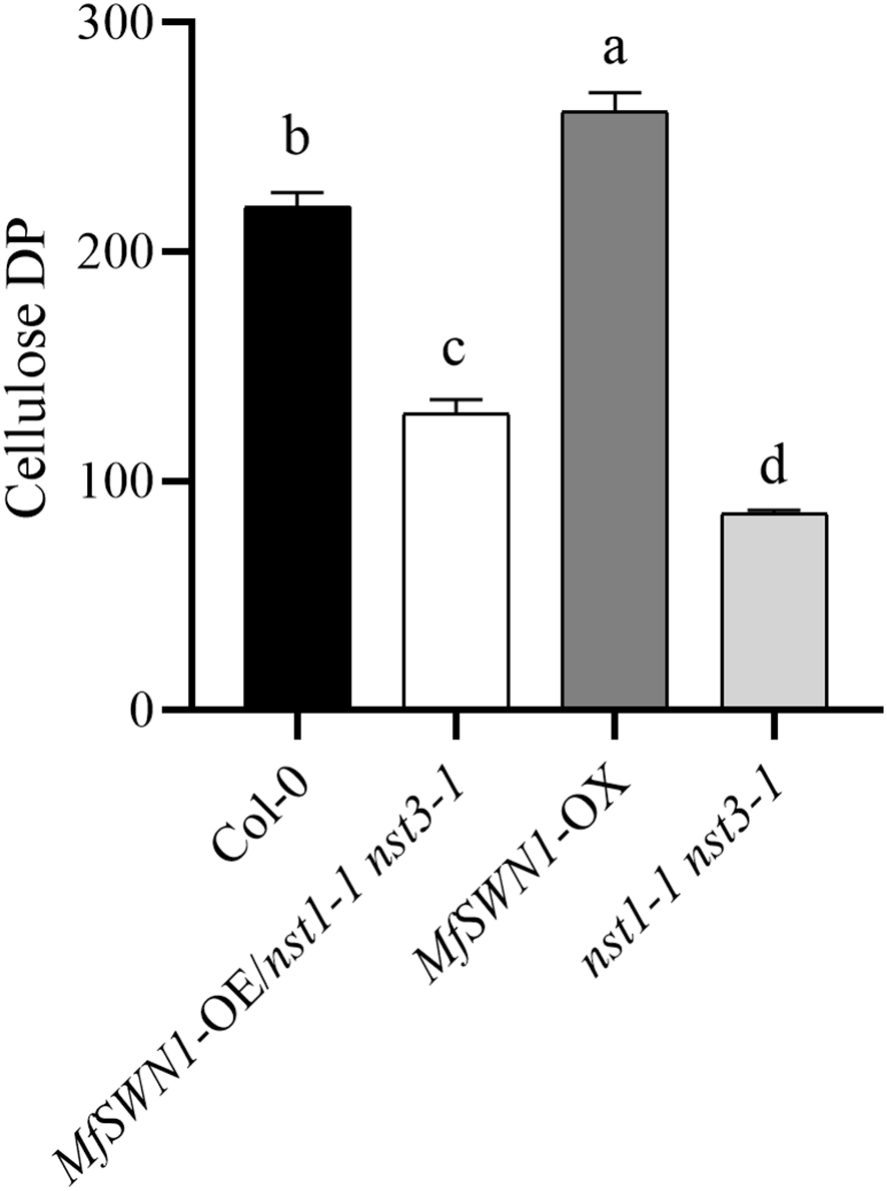
Determination of the DP value in crude cell walls from the stems of 6-week-old *Arabidopsis* plants. Different lowercase letters indicate significant differences among genotypes (*P* < 0.05).

DP was subsequently evaluated using the viscosity method. Consistent with the crystallinity analysis, DP values exhibited a similar trend: *MfSWN1*-OX plants displayed the highest DP, followed by Col-0, *MfSWN1*-OE/*nst1–1 nst3–1* plants, and *nst1–1 nst3–1* mutants, with all differences being statistically significant (**Figure 7**). These findings suggest that *MfSWN1* promotes the deposition of cellulose with both elevated CrI and an increased DP within SCWs. While CrI and DP are critical determinants of overall cell wall architecture, the scope of this study was primarily directed toward the structural characterization of SCWs.

## 4 Discussion

Plant organs are composed of tightly coordinated cell types that form rigid, mechanically integrated structures. During development, the geometric morphology of these cells plays a decisive role in determining plant growth patterns and mechanisms of environmental response (Dash et al., 2025; Silveira et al., 2025). Mechanical stresses arising from differences in growth rates between adjacent cell types propagate throughout the entire organ. The resulting activation of mechanosensitive signaling pathways can further alter nuclear morphology, and participate in the regulation of gene expression, cell fate determination, growth and development, organ formation, and stress responses (Agarwal and Zaidel-Bar, 2021; Komeda, 2004). In this study, the overexpression of *MfSWN1* led to enlarged, wrinkled rosette leaves and delayed bolting. NAC transcription factors associated with SCW biosynthesis are not classically recognized as regulators of floral pathways. Therefore, the delayed bolting observed in the *MfSWN1*-OX plants is likely an indirect developmental consequence of enhanced SCW deposition, rather than a direct regulatory effect on the floral transition itself (Mitsuda et al., 2007, 2005; Nakano et al., 2015). Enhanced deposition of lignocellulosic polymers can redirect carbon allocation from primary metabolism toward structural biomass production, thereby influencing vegetative growth and developmental timing (Nakano et al., 2015). Furthermore, increased secondary wall thickness alters the mechanical properties of plant tissues-such as stiffness and tensile strength-which subsequently affect organ development and growth dynamics (Mitsuda et al., 2007). Mutations in SCW-related NAC factors are known to impact reproductive tissue structure; for instance, the *Arabidopsis swn-1 clf-50* double mutant displays defective pollen wall development (Zhao et al., 2019). Similarly, various NAC transcription factors govern plant growth and tissue differentiation via their regulatory roles in SCW biosynthesis (Hussey et al., 2013; Zhong et al., 2007), suggesting that changes in SCW formation impact reproductive tissue morphology through structural mechanisms.

Genes involved in cellulose, hemicellulose, and lignin biosynthesis function coordinately during SCW formation (McFarlane et al., 2014). Specifically, *MYB46* and *MYB83*, two direct downstream targets of NAC transcription factors, serve as key regulators of the transcriptional network responsible for the control of SCW formation in vascular tissues (Zhong et al., 2010a; Zhong et al., 2011). *CesA4* encodes a cellulose synthase related to SCW synthesis (Ha et al., 2002). Xylan, the major hemicellulosic polysaccharide in dicot SCW, is synthesized by enzymes such as *IRX7* and *IRX8* (Scheller and Ulvskov, 2010). The *CCoAOMT1* participates in lignin biosynthesis, while *LAC4*, with high expression in vascular bundles and interfascicular fibers, may contribute to lignin polymerization (Berthet et al., 2011). Here, compared with Col-0 and *MfSWN1*-OE/*nst1–1 nst3–1* plants, the expression levels of these genes were significantly reduced in the *nst1–1 nst3–1* mutants. On the contrary, compared with Col-0, *MfSWN1*-OE/*nst1–1 nst3–1* plants were detected with markedly upregulated expression of downstream SCW-related genes, which were mostly upregulated in *MfSWN1*-OX plants as well. This result may support disruption in the downstream transcriptional program responsible for SCW biosynthesis when upstream SWN1 regulators are deleted. Interestingly, in *MfSWN1*-OE/*nst1–1 nst3–1* plants, the expression levels of several downstream genes were equal to or higher than those in *MfSWN1*-OX plants for some targets. It may unveil distinct biological contexts of the two transgenic systems. In other words, the disrupted regulatory pathway may be restored by *MfSWN1*-OE/*nst1–1 nst3–1* in the mutant background, while SCW-related transcription may be enhanced by *MfSWN1*-OX in the wild-type background. In the context of *MfSWN1*-OE/*nst1–1 nst3-1*, *MfSWN1* may restore the disrupted NAC-MYB regulatory cascade, further activating downstream target genes efficiently. In contrast, strong expression in *MfSWN1*-OX plants may enhance SCW deposition, coupled with partial transcriptional saturation or feedback regulation, which may restrict further increase in some downstream genes. Existing evidence has revealed such context-dependent regulatory behavior in SCW biosynthesis-associated transcription factors similarly (Golfier et al., 2017; Nakano et al., 2015; Rao and Dixon, 2018).

The expression of *MfSWN1* would affect multiple structural components of the cell wall. In this study, the observed increases in cellulose, hemicellulose, and lignin may imply coordinated regulation of SCW biosynthesis, rather than independent changes in individual polymers. This coordinated regulation aligns with the hierarchical NAC-MYB transcriptional network to control SCW formation in prior research (McFarlane et al., 2014). As a highly coordinated developmental process, the formation of SCW involves multiple layers of transcriptional regulation and metabolic integration. SWNs and other NAC transcription factors may be key determinants of this regulatory hierarchy, which may activate a cascade of MYB transcription factors and downstream structural genes related to cellulose, hemicellulose, and lignin biosynthesis. With advantages of facilitating coordinated synthesis, deposition, and assembly of cell wall polymers, this hierarchical network enables plants to produce mechanically robust tissues capable of providing structural support and efficient vascular transport (McFarlane et al., 2014). Previous studies have demonstrated strong structural interdependence between cellulose, hemicellulose, and lignin during cell wall assembly (Moneo-Sánchez et al., 2020). Consistently, the coordinated changes in cellulose, hemicellulose, and lignin in this study may cue an integrated transcriptional regulation of SCW biosynthesis, rather than independent alterations in individual SCW polymers. Collectively, *MfSWN1* may act as a key upstream regulator coordinating SCW biosynthesis and structural organization. Spatial cell biology *in situ* refers to integrative approaches that preserve whole organs or tissues in three dimensions and capture multiple regulatory layers within their native context. This method combines quantitative analysis of nuclear and chromatin structure, cellular geometry, protein localization, and protein-protein interactions, all based on high-resolution three-dimensional images. In future studies, the use of spatial cell biology approaches will help provide a comprehensive understanding of the mechanisms by which MfSWN1 may act as a key upstream regulator, coordinating SCW biosynthesis and structural organization. This approach will enable the detailed analysis and synchronization of the structural organization, chromatin dynamics, and positional signaling involved in the multi-layered transcriptional regulation and metabolic integration mechanisms underlying SCW formation (Pasternak and Yaroshko, 2026).

## 5 Conclusion

This study investigated in details the role of *MfSWN1* in regulating SCW development in *Arabidopsis thaliana*, highlighting the essential role of *MfSWN1* in SCW biosynthesis. Notably, *MfSWN1*-OE/*nst1–1 nst3–1* plants mainly exhibit functional restoration of the *nst1–1 nst3–1* mutants, while *MfSWN1*-OX plants display enhanced SCW formation in the Col-0 plants. Introduction of *MfSWN1* can restore stem lodging and indehiscent silique phenotypes of the *nst1–1 nst3–1* mutants, while its overexpression increases stem diameter, enhances lignification, alters silique morphology, and delays bolting. SCW-related genes, including *MYB46*, *MYB83*, *CesA4*, *LAC4*, *IRX7*, *IRX8*, and *CCoAOMT1*, are obviously upregulated in *MfSWN1*-OE/*nst1–1 nst3–1* plants relative to *nst1–1 nst3–1* mutants, with generally higher expressions than those in Col-0 plants, while most of these genes are also upregulated in *MfSWN1*-OX plants. *MfSWN1* overexpression also remarkably increases the content of cellulose, hemicellulose, and lignin. In addition, given the elevated DP and CrI, *MfSWN1* may also modulate cell wall structural organization. Overall, *MfSWN1* functions as an upstream regulator coordinating SCW biosynthesis and structural modification. Findings in this study may provide valuable reference for future studies on the genetic regulation of SCW formation and cell wall structural organization in the *Miscanthus* species.

## Data Availability

The original contributions presented in the study are included in the article/supplementary material. Further inquiries can be directed to the corresponding author.
